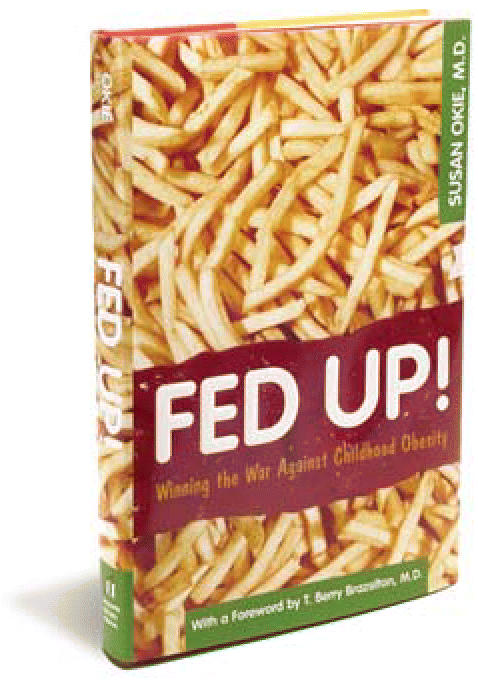# Fed Up! Winning the War Against Childhood Obesity

**Published:** 2006-05

**Authors:** Sandra G. Hassink

**Affiliations:** Sandra G. Hassink is the director of the Pediatric Weight Management Clinic at A.I. Dupont Hospital for Children in Wilmington, Delaware, which she began in 1988. This clinic uses a multidisciplinary; family based approach to obesity and takes care of children from infancy to young adulthood. Dr. Hassink is also a member of the American Academy of Pediatrics’ Task Force on Obesity

By Susan Okie

Washington, DC:Joseph Henry Press, 2005. 322 pp. ISBN: 0-309-09310-4, $27.95 cloth.

*Fed Up!* traces the obesity epidemic through the lives of children, families, teachers, and researchers. Examining the origins of the obesity epidemic, Susan Okie, a family physician and journalist, covers the distance between the “toxic environment” and the family. Sodas, juice, portions, fast food, television, school lunches, and sedentary lifestyles are all environmental factors that parents and families must confront in their efforts to stop the obesity epidemic. This book gives a closeup view of obesity’s effects on children’s lives by recounting children’s and adolescents’ daily struggles with body image, school snacks, the pull to inactivity, and families’ efforts to change lifestyle and behavior. In particular, Okie puts a face on the epidemic—the girls in the fifth-grade class and the articulate teen who struggles with weight. Tracing the “thrifty gene” hypothesis—that such a gene allows storage of calories during times of plenty that can be expended during famine—Okie discusses the interaction between genetics and environment and puts obesity research into action.

Chapters on pregnancy and the treatment of obesity are detailed and provide a start for parents who need an in-depth approach to the serious health implications and consequences of childhood obesity. Okie emphasizes that obesity is a family problem: “preventing unhealthy weight gain in American children will require adults to make profound changes in many of their own choices about diet, activity, and lifestyle.” Taking a detailed look at nutrition, Okie describes the rationale for a balanced diet, the dangers of fast food, and the slippery slope of increasing portion sizes, giving parents and families a clear picture of what has gone wrong in children’s nutritional environment. Likewise, a robust discussion of television provides evidence that decreased activity and increased snacking occurs when children and adolescents spend too much time in front of the screen. Okie suggests a variety of physical activities, both individual and group, that can take the place of sedentary screen time, and notes again that this change requires altering the entire family’s behavior. But school physical education, community and nonprofit organizations, and industry are also powerful influences.

Among the most interesting parts of the book are the interviews with obesity researchers. Insights into what prompted their dedication to solving the obesity puzzle to examples of their struggles and solutions in their own families provide readers with a unique perspective on the fight for a healthier environment and healthy children.

Schools are not spared in Okie’s search for solutions to obesity. Okie investigates the total school environment. School meals, snacks, soda machines, and the faulty economics of unhealthy eating are exposed. The ups and downs of school interventions are reviewed. Positive approaches to school change and the role of parents and teachers as advocates for change are refreshing. One example discussed is “school gardens and greenhouses, student-run fruit and vegetable stands, and farm-to-school programs,” such as the Berkeley, California, Edible Schoolyard.

In the strong chapter “Action for Healthy Communities,” Okie recommends community activities including political action to improve school environments and derail food marketing to children, the creation of safe play spaces in the community, and improved food choices in local markets. Such ideas give parents the beginning of an agenda for change.

*Fed Up!* is clear that “teaching children to make choices that add up to a healthier lifestyle requires a degree of sophistication that simply was not necessary for parents in the past.” This book will help parents and families close that gap and directs parents to look at their families, schools, communities, and beyond to improve the health of their children.

## Figures and Tables

**Figure:**